# Biological Rhythms and Preeclampsia

**DOI:** 10.3389/fendo.2013.00047

**Published:** 2013-04-08

**Authors:** Agnès J. Ditisheim, Charna Dibner, Jacques Philippe, Antoinette Pechère-Bertschi

**Affiliations:** ^1^Hypertension Unit, Division of Endocrinology, Diabetes, Hypertension and Nutrition, Department of Specialties of Medicine and Primary Care, Hôpitaux Universitaires de GenèveGeneva, Switzerland; ^2^Division of Endocrinology, Diabetes, Hypertension and Nutrition, Department of Specialties of Medicine, Hôpitaux Universitaires de GenèveGeneva, Switzerland

**Keywords:** circadian clock, pregnancy, preeclampsia, shift work, women health

## Abstract

The impact of impaired circadian rhythm on health has been widely studied in shift workers and trans-meridian travelers. A part from its correlation with sleep and mood disorders, biological rhythm impairment is a recognized risk factor for cardiovascular diseases and breast cancer. Preeclampsia is a major public health issue, associated with a significant maternal and fetal morbidity and mortality worldwide. While the risks factors for this condition such as obesity, diabetes, pre-existing hypertension have been identified, the underlying mechanism of this multi-factorial disease is yet not fully understood. The disruption of the light/dark cycle in pregnancy has been associated with adverse outcomes. Slightly increased risk for “small for gestational age” babies, “low birth weight” babies, and preterm deliveries has been reported in shift working women. Whether altered circadian cycle represents a risk factor for preeclampsia or preeclampsia is itself linked with an abnormal circadian cycle is less clear. There are only few reports available, showing conflicting results. In this review, we will discuss recent observations concerning circadian pattern of blood pressure in normotensive and hypertensive pregnancies. We explore the hypothesis that circadian misalignments may represent a risk factor for preeclampsia. Unraveling potential link between circadian clock gene and preeclampsia could offer a novel approach to our understanding of this multi-system disease specific to pregnancy.

## Introduction

Preeclampsia is a pregnancy-related disease. Its physiopathology, although not fully understood, probably involves multiple factors such as abnormal placentation, imbalance of angiogenesis regulators, and maternal immune maladaptation. It is thought to start early in pregnancy with a poor trophoblast invasion of maternal spiral arteries with a subsequent placental ischemia and insufficiency (Pennington et al., 2012). The maternal response, which is manifest in the second half of pregnancy, is marked by an increased systemic inflammatory response with high level of pro-inflammatory cytokines. The widespread endothelial dysfunction that ensues is responsible for the hypertension and proteinuria that characterize preeclampsia (Maynard and Karumanchi, 2011). Recently, it has been suggested that maternal hormonal disturbance arising from sleep deprivation or circadian rhythm disruption might impair fetal growth or lead to complications of pregnancy (Bonzini et al., 2011).

**Figure 1:**
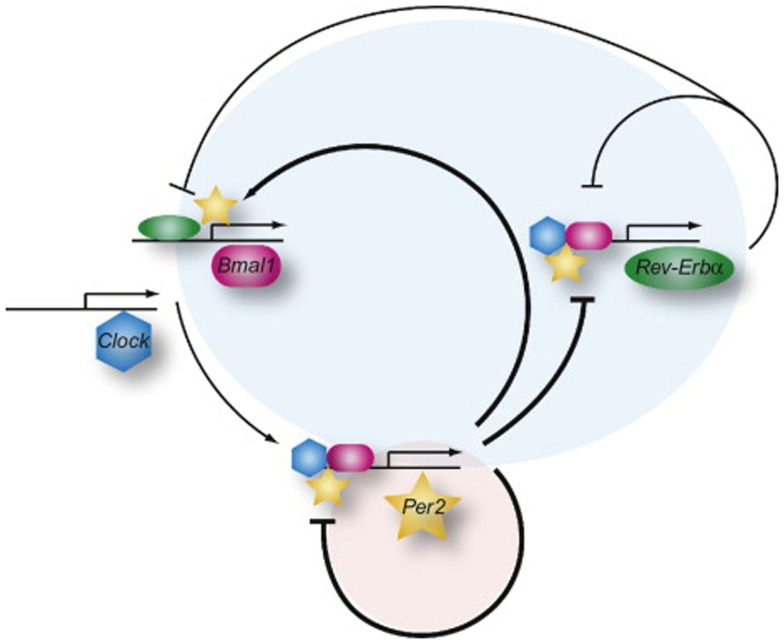
**The network of the mammalian molecular oscillator**. The core loop (red shaded circle), responsible for generating rhythms of about 24 h, is composed of activation of the *Per* genes by BMAL1 (red) and CLOCK (blue) and the increasing repression of *Per* gene expression by the accumulation of its own gene product. The feedback inhibition by the PER proteins is delayed by posttranslational modifications and interaction with the CRY proteins (not shown). In the stabilizing loop (blue circle), the *Rev-Erb*α gene (Reverse-erb alpha) is also activated by BMAL1 and CLOCK (not shown) and later on repressed by the PER proteins but immediately REV-ERBα starts to inhibit transcription of the *Bmal1* and *Clock* genes and of its own gene. PER2 can interact with REV-ERBα (or PPARα, not shown) to regulate the *Bmal1* gene. The overall organization of the network allows for a tight synchronization of the core and stabilizing loops. Adapted from Ripperger and Albrecht (2012).

Most physiological processes in mammals are synchronized with the day-night cycle through an internal mechanism called the circadian clock. This reflects the existence of underlying intrinsic clocks with near 24 h oscillation periods. The circadian system is organized in a hierarchic manner, with a master clock, located in the suprachiasmatic nucleus (SCN) in the hypothalamus, synchronizing billions of subsidiary oscillators located in peripheral tissues. The term “circadian” comes from the latin “circa diem” or “about a day,” meaning these clocks can only keep time approximately, and must be re-adjusted every day. The photoperiod is the most dominant environmental *Zeitgeber* (time giver) for the phase entrainment of circadian oscillators in all investigated organisms (Damiola et al., 2000; Reppert and Weaver, 2002). The SCN integrates luminous stimuli, through the retina and coordinates accordingly the peripheral « slave » clocks through direct hormonal and neuronal signals or via indirect cues such as body temperature or feeding time. The current molecular model for the generation of circadian oscillations is based on interlocked negative feedback loops in gene expression. In mammals, the two PAS-domain helix-loop-helix transcriptional activators BMAL1 (Brain and muscle ARNT-like protein 1) and CLOCK (Circadian locomotor output cycles kaput) form a heterodimer that activates Per (Period) and Cryptochrome (Cry) transcription (as illustrated in Figure 1; Ripperger and Albrecht, 2012). Once PER and CRY proteins accumulate, they form nuclear complexes that interfere with BMAL1/CLOCK mediated transactivation, and therefore inhibit their own transcription. This negative feedback loop generates cycles of around 24 h in gene expression (Ripperger and Schibler, 2006; Lowrey and Takahashi, 2011). In addition, posttranslational events such as the control of protein phosphorylation, sumoylation, acetylation, O-GlcNAcylation, degradation, and nuclear entry, contribute critically to the generation of daily oscillations in clock gene products (Cardone et al., 2005; Gallego and Virshup, 2007; Asher et al., 2008; Nakahata et al., 2008; Durgan et al., 2011; Reischl and Kramer, 2011; Kim et al., 2012). Central and peripheral clocks have a similar molecular makeup. Moreover, this rhythm generating circuitry is functional in most cell types, including primary and immortalized cell lines (Balsalobre et al., 1998; Nagoshi et al., 2004). The cellular clocks are cell-autonomous, self-sustained, and resilient to temperature changes and cell division (Dibner et al., 2010).

To the best of our knowledge, potential disruption of the circadian clockwork has scarcely been explored in preeclampsia. The first part of this review will focus on the clockwork machinery underlying placental function, a major player in the pathology of preeclampsia. We will then explore the indirect evidence of circadian misalignment in preeclampsia, such as disruption of the physiological circadian pattern of blood pressure (BP) and conclude with potential clinical implications, perspectives, and unsolved research questions.

## Clockwork and Placental Function

Little information is available regarding clock machinery function in the placenta. Frigato et al. investigated clock gene expression in human placenta. This study suggests a circadian expression pattern for Per2, Dec1, and DBP transcripts in *in vitro* synchronized HTR/SVneo cells established from a human extra villous trophoblast during the first trimester of pregnancy. Interestingly, the expression of these genes was preserved and exhibited even higher oscillation amplitude under hypoxic conditions, probably due to the similarities between extra villous trophoblast and tumor cells. In addition, the response of the circadian clock to hypoxia could represent an adaptive mechanism providing adequate vascularization to answer placental needs (Frigato et al., 2009). Expression of vascular endothelial growth factor (VEGF) has also been explored. VEGF oscillation started only 30 h following the synchronization stimulus (serum shock), and its circadian pattern remains questionable.

Two more studies reported the presence of canonical core clock genes in the rodent placenta. Endogenous levels of core clock transcripts Bmal1, Clock, Cry (Cry1-2), and Period (Per1-2) exhibited oscillatory patterns in the murine gravid uterus, placenta, and fetal membrane during the last third of gestation (Ratajczak et al., 2010). In line with these findings, the level of circadian bioluminescence reporter Per2:luciferase oscillated in these tissues. Interestingly the absolute expression levels of the core clock genes were upregulated 2 days before the parturition, suggesting that peripheral clocks in the reproductive tissues might be important during gestation. An additional study showed the expression of canonical core clock transcripts in two functionally distinct zones of rat placenta, the labyrinth (involved in maternal-fetal exchange) and the junctional zone (responsible for hormone production) (Wharfe et al., 2011). While all the assayed clock transcripts were expressed in both zones, their expression levels did not significantly vary around-the-clock. In the labyrinth zone, Clock, Per1, and Cry2 expression was higher compared to the junctional zone, while the opposite tendency was observed for Bmal1, Per2, Per3, and Cry1. A constant expression of VEGF over the circadian time points was also demonstrated in the placenta in this study.

The physiological significance of these findings needs to be further explored. Based on the two available *in vivo* studies in rodent models, it is difficult to conclude whether the core clock genes exhibit the oscillatory pattern in rodent placenta. However the study performed in HTR/SVneo human primary cells synchronized *in vitro* by serum shock (Frigato et al., 2009) strongly suggests the existence of the functional placental circadian clock. Moreover, this work and the study of rat placenta (Wharfe et al., 2011) suggests that placental angiogenic factors like VEGF might be candidates for the placental circadian clock regulation.

### Circadian expression of placental inflammatory mediators

Preeclampsia is characterized by a marked inflammatory state. Whether this is the result of a placental ischemia and reperfusion or an exaggerated maternal response to the allogenic fetus remains uncertain (Sibai et al., 2005).

In their review, Waddell et al. mention recent results suggesting that pro-inflammatory cytokines, particularly TNF-alpha, are expressed in the placenta according to a circadian pattern. This has also been observed for IL-6 and IL-1beta (Waddell et al., 2012). The significance of rhythmic expression of placental pro-inflammatory cytokines in physiological process such as parturition needs to be determined.

## Circadian Pattern of Blood Pressure: Indirect Evidence of the Potential Role of the Clock in Preeclampsia

### Circadian pattern of blood pressure in normal pregnancies

Arterial BP is one of the major physiological processes following a circadian pattern. In non-pregnant women, BP is highest early in the morning. It increases again in the afternoon or late evening and is at its lowest during the night, dipping to 10–20% less than day time values.

Normotensive pregnancies preserve the normal BP circadian pattern, but with a lower mean, as shown in the majority of the control groups of the studies summarized in Table 1. BP tends to decrease from the first trimester to the second trimester. It then rises again to reach a non-pregnant value near term. The most simple and clinical way to evaluate the nycthemeral pattern of the BP is to monitor the 24 h ambulatory BP, and calculate the nocturnal dip. In clinical research, more sophisticated chronobiological methods to evaluate oscillations of the BP are used. The circadian pattern of arterial pressure in physiologic pregnancies compared to the non-pregnant state display a less pronounced *midline estimating statistic of rhythm* (mesor). Mesoris the arithmetical mean of the measures of the biological variable, or the mean level of the rhythm (Cugini et al., 1992; Benedetto et al., 1996). As mesoris the measurable expression of the tonic level for a given periodic function, Cugini et al. suggest that the differences seen in the 24 h BP pattern between the pregnant and the non-pregnant state lies in its tonic regulation. This might be a mechanism that reduces the baric impact of pregnancy and protects the arterial vascular system.

**Table 1:** **Summary of the main studies on BP and circadian pattern during normal and abnormal pregnancies**.

Reference	*n*	Methods	Results	Comments
Seligman (1971)	*n* = 30 (10 normal BP, 10 HT, 10 PE)	Automatic recorder (24 h) Inpatients	Greater fall at night in HT Reduced fall at night in PE	Characteristics of the subject included are lacking (age, parity, weeks gestation)
				Results and figures not detailed for every subject
Redman et al. (1976)	*n* = 19 [3 severe PE, 4 mild HT, 12 primigravida w/mild HT (6 on methyldopa, 6 no treatment)]	Automatic recorder (48 h) Inpatients, bedrest At 24 and 36 w.g.	Reversed rhythm in severe PE Normal pattern in mild HT	Characteristics of the women included are lacking (age, parity) Results and figures not detailed for every subject
Sawyer et al. (1981)	*n* = 45 (15 normal BP, 15 chronic HT, 15 mild PE), third trimester	DINAMAP (24 h) Inpatients	Blunted fall at night in PE No diurnal variation in two severe PE (excluded from analysis)	Characteristics of the women included are lacking (age, parity not precised except for PE)
Beilin et al. (1983)	*n* = 31 [10 normal BP, 13 HT (antenatal BP normal), 8 PE]	DINAMAP (24 h) Inpatients	Same circadian rhythm in normo and hypertensive groups	Two HT and three PE under treatment when studied
	>26 w.g.		No fall in BP in all PE, with four nocturnal HT	PE older, multiparous
Cugini et al. (1992)	*n* = 60 (30 non-pregnant, 30 pregnant women)	ABPM (24 h) At 8–10, 18–20, and 32–34w.g.	Reduced means BP for gestational HT. Values tend to increase in the second and third trimester	
Benedetto et al. (1996)	*n* = 212 (73 controls, 48 GH, 38 PE, 53 mild to moderate HT)	DINAMAP (24 h) Inpatients	BP oscillations less pronounced in GH and PE	Circadian parameters obtained by single cosinor method
	Singleton pregnancy	At 8–16, 20–25, 28–35, 36–40 w.g.	Severity of HT favor the loss of diurnal rhythm, especially in PE	
Halligan et al. (1996)	*n* = 48 (24 normal BP, 24 PE), 35 w.g.	ABPM (24 h)	Blunted BP nocturnal fallin PE. Inversely related to increase in BP mean	Preliminary, cross-sectional study Comparison of means and gradients
Tranquilli et al. (1996)	*n* = 114 (Normal BP, 36 IUGR, birthweight < fifth percentile, 78 normal IUG and birthweight	ABPM (24 h) At 24–28 w.g.	BP significantly elevated in IUGR although still in normal range	
Ayala et al. (1997)	*n* = 113 (71 uncomplicated pregnancy, 28 GH, 14 PE)	ABPM (48 h) Every 4 weeks after first obstetric visit	Significant difference in BP between healthy and complicated pregnancies present in the first 14 w.g.	
Olofsson and Poulsen (1997)	*n* = 56 GH (of which 34 PE)	ABPM (24 h) Inpatients (range 23–40 weeks gestation)	12 SBP reversed rhythm, of which 10 PE. Associated with a more severe PE, smaller BP variation, maintenance of fetal growth, higher birthweight	No control group
Stoynev et al. (1999)	*n* = 31 pregnant women at low risk for HT	Automatic recorder (48 h) Inpatients	HR mesor increased in middle and late pregnancy. BP mesor unchanged	
Hermida et al. (2000)	*n* = 202 (124 normal pregnancy, 55 GH, 23 PE)	ABPM (48 h) Every 4 weeks after first obstetric visit until delivery	Difference in circadian variability present in the first 14 w.g.	Circadian parameters established by population multiple component-analysis
Brown et al. (2001)	*n* = 158 (63 PE, 68 GH, 27 HT)	ABPM (24 h) Inpatients or outpatients	High prevalence of nocturnal HT in PE and GH/essential HT	
Hermida et al. (2003)	*n* = 403 (235 uncomplicated pregnancies, 128 GH, 40 PE)	ABPM (48 h) Every 4 weeks after first obstetric visit until delivery	Difference in the circadian variability already in first trimester	Circadian parameters established by population multiple component-analysis
Hermida et al. (2004)	*n* = 434 (245 uncomplicated pregnancies 140 GH, 49 PE)	ABPM (48 h) Every 4 weeks after first obstetric visit until delivery	Significant difference in the 14 first w.g. between normal and complicated pregnancies	Circadian parameters established by population multiple component-analysis
				Comparison of the 24 h mean of pulse pressure

*BP, blood pressure; SBP, systolic blood pressure; DBP, diastolic blood pressure; ABPM, ambulatory blood pressure monitoring; HT, hypertension; GH, gestational hypertension; IUGR, intrauterine growth retardation; w.g, weeks gestation; DINAMAP, device for indirect non-invasive automatic mean arterial pressure*.

### Circadian variation of blood pressure in preeclampsia

Blood pressure diurnal variation in complicated pregnancies has been studied since the 1950s in an attempt to understand the underlying mechanisms of hypertensive disease, set norms for gestational BP, and optimize screening tools for early detection.

Before the arrival of the chronobiological method of analysis, BP variation was assessed by comparing means and gradients, or by calculating the percentage of diurnal variation. Studies on the subject are rather limited, with little or no detailed figures or characteristics of the population included, making the validity of the results questionable. However, these early studies reveal important and interesting observations regarding possible BP rhythmicity perturbations in complicated pregnancies. Table 1 summarizes most of the published data.

When compared to normotensive pregnancies, pregnant women with essential hypertension preserve a normal circadian variation of BP but with a greater fall in BP at night (Seligman, 1971; Redman et al., 1976; Sawyer et al., 1981). These intriguing results need to be confirmed in larger studies.

In preeclampsia, evolution of BP throughout pregnancy is distinct. Contrary to physiologically normal pregnancies, BP does not decrease in the first trimester, but instead remains stable during the first half of pregnancy and continuously increases until delivery (Hermida et al., 2000). More interestingly, several studies show consistent alterations in circadian rhythm. In preeclampsia, the nocturnal decrease of BP is blunted and there is less variation among BP circadian values (Seligman, 1971; Sawyer et al., 1981; Beilin et al., 1983; Halligan et al., 1996). Later chronobiological studies found concordant results, with a decrease in the oscillatory amplitude of BP (i.e., the extent of oscillatory wave from MESOR) in the two last trimesters of hypertensive and preeclamptic pregnancies (Benedetto et al., 1996; Hermida et al., 2003).

In severe cases, patients may even display a reversed rhythm with maximal BP values occurring at night (Redman et al., 1976; Sawyer et al., 1981; Beilin et al., 1983). The reason for this blunted or even reversed circadian pattern in preeclampsia is unknown.

Beilin et al. explored the correlation of pressor hormones with the measured arterial pressure in three groups of 15 normotensive, chronic hypertensive, and preeclamptic pregnancies. They observed a suppression of plasma renin activity and angiotensin II levels during the day and a loss of the diurnal pattern for angiotensin II in preeclamptic women. A reversed pattern for the norepinephrine sulfate level was found as well as a suggestion of loss of the midnight drop in the free adrenaline plasma level. In the preeclamptic group, four patients had nocturnal hypertension and showed the lowest levels of plasma renin activity and angiotensin II. Absence of an angiotensin II, adrenaline, or noradrenaline drop at night in subjects with increased vascular reactivity could contribute to their nocturnal hypertension, with a direct negative feedback of the high BP on the renal vascular barostat (Beilin et al., 1983).

Olofsson and Poulsen also found a reversed BP pattern in 12 of the 56 women investigated for pregnancy-induced hypertension of which, 36 had preeclampsia. As expected, reversed BP rhythm was associated with the more severe form of preeclampsia (Olofsson and Poulsen, 1997). Surprisingly, the study showed that women with reversed rhythm delivered larger babies. The authors hypothesized that a more stable BP, prolonged over the night, maintained higher placental perfusion that was beneficial for the fetus.

Another pathophysiologic mechanism evoked by some authors to explain the non-dipper pattern in the general hypertensive population is linked to the pressure-natriuresis curve described by Guyton et al. (1972). Some individuals, probably sub-groups, such as menopausal women, that are salt-sensitive or with slight renal alterations need to have an increased BP at nighttime in order to excrete an excess of sodium retained during the day (Pechere-Bertschi and Burnier, 2004). Preeclamptic women are rather hypovolemic despite the edematous state, with an increased sodium retention in the extravascular compartment as well as a strong vasoconstrictive status. Non-dipper status observed in preeclampsia could be a compensative mechanism to oppose the increased sodium retention as well as an adaptive mechanism to preserve fetal perfusion.

## Clinical Implications

### Circadian pattern of blood pressure as a predictor of preeclampsia

Identifying high-risk patients by early recording of a 24 h ambulatory BP monitoring (ABPM) and analyzing changes in the circadian pattern of BP, could be an elegant, non-invasive, and economic way of predicting hypertensive complications of pregnancy.

Screening for gestational hypertension (GH) is usually made by office BP measurement. ABPM is commonly used as a confirmative diagnostic tool, when an isolated abnormal office BP is measured. However, subtle differences in the BP pattern, although well within the normal range, can be detected early in the course of pregnancies that will later develop hypertensive complications (Ayala and Hermida, 2013).

#### Circadian variability and pulse pressure

In a large prospective study, the Hermida et al. (2003) group explored the circadian variability of BP as an early predictor of hypertensive complications of pregnancy. The authors showed a highly statistically significant difference in the circadian variability in BP between normotensive and complicated pregnancies, already detectable in the 14 first weeks of gestation. Amplitude was also significantly higher in the first and second trimesters of later complicated pregnancies, with a tendency to decrease in the second and third trimesters, as mentioned earlier, mainly due to a blunted night drop in preeclamptic pregnancies.

Using the same study design, Hermida et al. compared the 24 h mean of pulse pressure (PP) between healthy and complicated pregnancies. PP is the measure of the pulsatile component of BP which represents a marker of the cardiovascular risk in the general population. A significant elevation in 24 h mean PP sampled by ABPM was present in the first 14 weeks of gestation. It has to be stressed that this subtle difference in later hypertensive pregnancies appears while BP is still within the normal range (Hermida et al., 2004).

#### Hyperbaric index

Hyperbaric index (HBI) refers to the area of BP excess above the upper limit value. It has been evaluated as a diagnostic tool for GH. Hermida et al. validated this approach prospectively in 1998 and found a test sensitivity of 93% for women screened in the first trimester, and of 99% in the third trimester. Positive and negative predictive values averaged 96% in all trimester. The HBI was abnormal 23 weeks before manifest hypertension (Hermida et al., 1998). In preeclampsia, HBI is significantly higher after 20 weeks gestation (Hermida and Ayala, 2002).

### Implication of chronotherapy in the prevention of preeclampsia using low-dose aspirin

Administration of low-dose aspirin has been shown to reduce the overall risk of preeclampsia (Sibai et al., 1993). This protective property is more pronounced if aspirin is taken before or at 16 weeks of gestation with a 89% reduction of preterm preeclampsia (Roberge et al., 2012b) and a *ca*. 90% decrease in relative risk of severe preeclampsia (Roberge et al., 2012a).

Preeclampsia is associated with an imbalanced production of prostacyclin (a vasodilator) and thromboxane A (a vasoconstrictor and platelet aggregator), with a subsequent arteriolar vasoconstriction and coagulation disorder. The protective effect of aspirin is thought to be due to a selective decrease in thromboxane A production with no influence on the prostacyclin level (Sibai et al., 1993, 2003).

Amongst other properties, low-dose aspirin has been shown to reduce BP. This action might be linked the antioxidative effect of aspirin, with a reduction in vascular production of superoxide and an increased release of nitric oxide (NO) (Hermida et al., 2005b). Moreover, aspirin has been shown to influence secretion of pressor hormones such as renin, aldosterone, cortisol, and catecholamine (Snoep et al., 2009). Interestingly, this phenomenon seems to be influenced by ingestion time. When taken at bedtime, aspirin is effective in lowering BP whereas this effect is lost when taken in the morning (Hermida et al., 2005a). Although not fully understood, this might be related to the circadian secretion pattern of BP regulators. As explored by Snoep et al. (2009) aspirin decreases the nocturnal rise in activity of the renin-angiotensin-aldosterone system and in cortisol and catecholamine levels when taken at bedtime, but not in the morning. Another mechanism could involve enhanced release of NO during the night, as exemplified by Hermida et al. in a study comparing BP response to aspirin in non-dipper hypertensive patients and dipper patients. Non-dipper status is characterized by a reduced NO vascular production. When administered at night, aspirin not only produced a significant reduction in BP, but the reduction in the non-dipper group was twice that observed in the dipper group (Hermida et al., 2005b).

This has been observed in normotensive, pre-hypertensive, and mild hypertensive subjects, as well as in pregnant women at higher risk for GH or preeclampsia (Hermida et al., 1997; Ayala et al., 2013).

Ayala et al. conducted a randomized, placebo-controlled trial on 350 women with an elevated risk of GH or preeclampsia, to compare the effect of low-dose aspirin, when ingested at different times of the day. The primary outcome was serious adverse event, defined as preeclampsia, preterm delivery, intrauterine growth retardation, and stillbirth. An additional endpoint was the composite of these adverse events plus GH (Ayala et al., 2013). In concordance with previous studies, women in the aspirin group had a significantly lower incidence of serious adverse outcomes with or without GH. Reduction in risk of preeclampsia was individually documented in the aspirin group (*p* = 0.41) with a number needed to treat (NNT) of 16. This reduction in risk was greater when aspirin was taken in the evening or at bedtime compared to the placebo group plus the aspirin ingested at awakening group (*p* < 0.01) and the NNT halved to 8.

In line with previous studies, there was no significant difference in BP between placebo and aspirin ingested in the morning. There was a significant decrease in BP when aspirin was taken 8 h after awakening, and to a greater extent when it was taken at bedtime.

Administration of low-dose aspirin at bedtime represents an easy and cost effective prophylaxia for women with a high risk of developing preeclampsia.

## Misalignment of Biological Rhythm and Risk of Preeclampsia

As introduced earlier, many physiological processes occur in phase with environmental cues or *Zeitgebers*, the most important one being the daily change in light intensity. Various occupations require night work and iterative crossing of time zones. Healthcare professionals and security workers have to work night shifts, whereas flight attendants and pilots are regularly confronted with jetlag.

Disruption of the circadian rhythm has been associated with impaired reproductive function and adverse pregnancy outcomes. Women working night shifts or transmeridian travelers might be at increased risk for spontaneous abortion, subfecundity, as well as giving birth to premature babies or low birth weight babies (Mahoney, 2010).

While physically demanding jobs have been associated with a slight increase in GH and preeclampsia (Mozurkewich et al., 2000), very few data are available on a potential link between shift work and preeclampsia.

A Norwegian cross-sectional study conducted in 1989 showed no association between shift work and prevalence of preeclampsia (OR 1.3, CI 0.8–1.9) in the overall population studied (Wergeland and Strand, 1997). However, authors found an interaction with parity and prevalence of preeclampsia that was increased among parous women working shifts (OR 2.0, 95% CI 1.1–3.6).

Haelterman et al. (2007) performed a case-control study in women from six region of Quebec between 1997 and 1999. Evening or night hours were not associated with an increased prevalence of preeclampsia (1–6 weekly evening work hours: OR 1.0, 95% CI 0.6–1.7, 7–32 weekly evening work hours: OR 1.1, 95% CI 0.7–1.9, ≥1 weekly night work hours: OR 1.0 95% CI 0.5–2.0).

In their large cross-sectional study, Chang et al. (2010) did not show any association between shift work and preeclampsia among Taiwanese women between June 2005 and July 2006 (adjusted OR 0.96, 95% CI 0.59–1.57).

These three studies did not bring convincing evidence that shift work increases the risk of preeclampsia.

## Discussion and Perspective

Recently, there has intense research addressing the relationship between the circadian internal clock genes and some aspects of cardiovascular and general health. Preeclampsia can be considered as a stress test for cardiovascular diseases (Sattar and Greer, 2002), and there is some evidence both in animal and human experiments that circadian misalignment may be associated with this pathology.

Early in pregnancy, some clock genes and cytokines are expressed in the placenta according to a circadian pattern, with different oscillation amplitudes under pathologic conditions (Frigato et al., 2009; Wharfe et al., 2011; Waddell et al., 2012).

In both normotensive and hypertensive populations, a non-dipper status of the nocturnal BP has been linked to an increased risk of target organ damage, microalbuminuria, adverse renal outcome, and mortality. A non-dipping pattern of BP is associated with an augmented risk of developing preeclampsia. Other variables of BP, like increased variability and PP, have been related to abnormal pregnancies. It is unknown if the non-dipping status is a compensative mean to excrete the higher rate of sodium retained during the daytime in abnormal pregnancy. Indeed, absence or blunted decrease of the BP during nighttime has been associated with salt sensitivity of the BP, which is a well-known cardiovascular risk factor. In animal models, mice lacking the core clock components Cry1 and Cry2 (Cry-null mice), show salt-sensitive hypertension due to abnormally high synthesis of aldosterone (Okamura et al., 2011). As mentioned by Olofsson and Poulsen (1997) an adaptive mechanism to preserve fetal perfusion should also be considered.

Concordant data show that when started early in pregnancy, low-dose aspirin is effective in reducing the overall risk of preeclampsia, but especially the risk of preterm and severe preeclampsia (Roberge et al., 2012a,b). Based on chronotherapeutic studies, bedtime rather than morning time ingestion has been shown to reduce BP and seems to considerably improve the prophylactic property of aspirin (Ayala et al., 2013). This simple, safe, and cost effective way to improve the outcome of some high-risk pregnancies should encourage clinicians to prescribe this medication in the evening.

High-risk pregnancies are currently identified according to personal risk factors. Development of an efficient screening tool for early detection of high-risk pregnancies, especially among healthy nulliparous, has yet to be achieved. Analysis of various parameters recorded by ABPM allows detection of differences in BP patterns very early in pregnancies that are later complicated by preeclampsia. However, these differences remain subtle and within the normally accepted physiological range. Setting of ABP normal values for each trimester and a standardized analysis of BP circadian pattern convenient to clinical use are still required. Moreover, there are concerns about the reproducibility of the circadian pattern by ABPM, as tolerance to the device varies among patients and can disrupt sleep. The validity of ABPM still has to be confirmed before implementing it in clinical practice for early detection of hypertensive diseases of pregnancy.

Many professional fields demand an around-the-clock activity, exposing pregnant workers to artificial lights, irregular and night shifts, as well as disturbed sleep habits. Shift work has been identified as a risk factor for diseases such as cancer (Straif et al., 2007). However, very few cross-sectional studies explored disruption of circadian rhythm as an occupational risk factor for preeclampsia, with no persuasive evidence. Considering that modern society increasingly upsets our biological rhythm, this question has to be addressed for preeclampsia, a major public health concern.

Altogether, this review highlights the need for further genetic, experimental, and clinical studies to explore the rhythmicity of placental function, develop powerful screening tools for preeclampsia, and verify whether a disrupted circadian rhythm represents an occupational risk factor for working pregnant women.

## Conflict of Interest Statement

The authors declare that the research was conducted in the absence of any commercial or financial relationships that could be construed as a potential conflict of interest.

## References

[B1] Asher G. Gatfield D. Stratmann M. Reinke H. Dibner C. Kreppel F. (2008). SIRT1 regulates circadian clock gene expression through PER2 deacetylation. Cell 134, 317–328 10.1016/j.cell.2008.06.050 18662546

[B2] Ayala D. E. Hermida R. C. (2013). Ambulatory blood pressure monitoring for the early identification of hypertension in pregnancy. Chronobiol. Int. 30, 233–259 10.3109/07420528.2012.701489 23006127

[B3] Ayala D. E. Hermida R. C. Mojon A. Fernandez J. R. Iglesias M. (1997). Circadian blood pressure variability in healthy and complicated pregnancies. Hypertension 30, 603–610 10.1161/01.HYP.30.3.603 9322989

[B4] Ayala D. E. Ucieda R. Hermida R. C. (2013). Chronotherapy with low-dose aspirin for prevention of complications in pregnancy. Chronobiol. Int. 30, 260–279 10.3109/07420528.2012.701489 23004922

[B5] Balsalobre A. Damiola F. Schibler U. (1998). A serum shock induces circadian gene expression in mammalian tissue culture cells. Cell 93, 929–937 10.1016/S0092-8674(00)81199-X 9635423

[B6] Beilin L. J. Deacon J. Michael C. A. Vandongen R. Lalor C. M. Barden A. E. (1983). Diurnal rhythms of blood pressure, plasma renin activity, angiotensin II and catecholamines in normotensive and hypertensive pregnancies. Clin. Exp. Hypertens. B 2, 271–293 6347445 10.3109/10641958309006086

[B7] Benedetto C. Zonca M. Marozio L. Dolci C. Carandente F. Massobrio M. (1996). Blood pressure patterns in normal pregnancy and in pregnancy-induced hypertension, preeclampsia, and chronic hypertension. Obstet. Gynecol. 88, 503–510 10.1016/0029-7844(96)00217-7 8841207

[B8] Bonzini M. Palmer K. T. Coggon D. Carugno M. Cromi A. Ferrario M. M. (2011). Shift work and pregnancy outcomes: a systematic review with meta-analysis of currently available epidemiological studies. BJOG 118, 1429–1437 10.1111/j.1471-0528.2011.03066.x 21790955 PMC3388382

[B9] Brown M. A. Davis G. K. McHugh L. (2001). The prevalence and clinical significance of nocturnal hypertension in pregnancy. J. Hypertens. 19, 1437–1444 10.1097/00004872-200108000-00012 11518852

[B10] Cardone L. Hirayama J. Giordano F. Tamaru T. Palvimo J. J. Sassone-Corsi P. (2005). Circadian clock control by SUMOylation of BMAL1. Science 309, 1390–1394 10.1126/science.1110689 16109848

[B11] Chang P. J. Chu L. C. Hsieh W. S. Chuang Y. L. Lin S. J. Chen P. C. (2010). Working hours and risk of gestational hypertension and pre-eclampsia. Occup. Med. (Lond.) 60, 66–71 10.1093/occmed/kqp119 19700491

[B12] Cugini P. Di Palma L. Battisti P. Leone G. Pachi A. Paesano R. (1992). Describing and interpreting 24-hour blood pressure patterns in physiologic pregnancy. Am. J. Obstet. Gynecol. 166, 54–60 1733219 10.1016/0002-9378(92)91829-y

[B13] Damiola F. Le Minh N. Preitner N. Kornmann B. Fleury-Olela F. Schibler U. (2000). Restricted feeding uncouples circadian oscillators in peripheral tissues from the central pacemaker in the suprachiasmatic nucleus. Genes Dev. 14, 2950–2961 10.1101/gad.183500 11114885 PMC317100

[B14] Dibner C. Schibler U. Albrecht U. (2010). The mammalian circadian timing system: organization and coordination of central and peripheral clocks. Annu. Rev. Physiol. 72, 517–549 10.1146/annurev-physiol-021909-135821 20148687

[B15] Durgan D. J. Pat B. M. Laczy B. Bradley J. A. Tsai J. Y. Grenett M. H. (2011). O-GlcNAcylation, novel post-translational modification linking myocardial metabolism and cardiomyocyte circadian clock. J. Biol. Chem. 286, 44606–44619 10.1074/jbc.M111.278903 22069332 PMC3247942

[B16] Frigato E. Lunghi L. Ferretti M. E. Biondi C. Bertolucci C. (2009). Evidence for circadian rhythms in human trophoblast cell line that persist in hypoxia. Biochem. Biophys. Res. Commun. 378, 108–111 10.1016/j.bbrc.2008.11.006 19000901

[B17] Gallego M. Virshup D. M. (2007). Post-translational modifications regulate the ticking of the circadian clock. Nat. Rev. Mol. Cell Biol. 8, 139–148 10.1038/nrm2106 17245414

[B18] Guyton A. C. Coleman T. G. Cowley A. V. Jr. Scheel K. W. Manning R. D. Jr. Norman R. A. Jr. (1972). Arterial pressure regulation. Overriding dominance of the kidneys in long-term regulation and in hypertension. Am. J. Med. 52, 584–594 10.1016/0002-9343(72)90050-2 4337474

[B19] Haelterman E. Marcoux S. Croteau A. Dramaix M. (2007). Population-based study on occupational risk factors for preeclampsia and gestational hypertension. Scand. J. Work Environ. Health 33, 304–317 10.5271/sjweh.1147 17717623

[B20] Halligan A. Shennan A. Lambert P. C. De Swiet M. Taylor D. J. (1996). Diurnal blood pressure difference in the assessment of preeclampsia. Obstet. Gynecol. 87, 205–208 10.1016/0029-7844(95)00379-7 8559524

[B21] Hermida R. C. Ayala D. E. (2002). Prognostic value of office and ambulatory blood pressure measurements in pregnancy. Hypertension 40, 298–303 10.1161/01.HYP.0000028978.99648.D0 12215470

[B22] Hermida R. C. Ayala D. E. Calvo C. Lopez J. E. (2005a). Aspirin administered at bedtime, but not on awakening, has an effect on ambulatory blood pressure in hypertensive patients. J. Am. Coll. Cardiol. 46, 975–983 10.1016/j.jacc.2004.08.071 16168278

[B23] Hermida R. C. Ayala D. E. Calvo C. Lopez J. E. Mojon A. Rodriguez M. (2005b). Differing administration time-dependent effects of aspirin on blood pressure in dipper and non-dipper hypertensives. Hypertension 46, 1060–1068 10.1161/01.HYP.0000172623.36098.4e 16087788

[B24] Hermida R. C. Ayala D. E. Iglesias M. (2004). Differences in circadian pattern of ambulatory pulse pressure between healthy and complicated pregnancies. Hypertension 44, 316–321 10.1161/01.HYP.0000139915.66288.b8 15289468

[B25] Hermida R. C. Ayala D. E. Iglesias M. Mojon A. Silva I. Ucieda R. (1997). Time-dependent effects of low-dose aspirin administration on blood pressure in pregnant women. Hypertension 30, 589–595 10.1161/01.HYP.30.6.1531 9322987

[B26] Hermida R. C. Ayala D. E. Mojon A. Fernandez J. R. Alonso I. Aguilar M. F. (2003). Differences in circadian blood pressure variability during gestation between healthy and complicated pregnancies. Am. J. Hypertens. 16, 200–208 10.1016/S0895-7061(03)00202-4 12620698

[B27] Hermida R. C. Ayala D. E. Mojon A. Fernandez J. R. Alonso I. Silva I. (2000). Blood pressure patterns in normal pregnancy, gestational hypertension, and preeclampsia. Hypertension 36, 149–158 10.1161/01.HYP.36.2.149 10948070

[B28] Hermida R. C. Ayala D. E. Mojon A. Fernandez J. R. Silva I. Ucieda R. (1998). Blood pressure excess for the early identification of gestational hypertension and preeclampsia. Hypertension 31, 83–89 10.1161/01.HYP.31.1.83 9449396

[B29] Kim E. Y. Jeong E. H. Park S. Jeong H. J. Edery I. Cho J. W. (2012). A role for O-GlcNAcylation in setting circadian clock speed. Genes Dev. 26, 490–502 10.1101/gad.195248.112 22327476 PMC3305986

[B30] Lowrey P. L. Takahashi J. S. (2011). Genetics of circadian rhythms in mammalian model organisms. Adv. Genet. 74, 175–230 10.1016/B978-0-12-387690-4.00006-4 21924978 PMC3709251

[B31] Mahoney M. M. (2010). Shift work, jet lag, and female reproduction. Int. J. Endocrinol. 2010, 813764 20224815 10.1155/2010/813764 PMC2834958

[B32] Maynard S. E. Karumanchi S. A. (2011). Angiogenic factors and preeclampsia. Semin. Nephrol. 31, 33–46 10.1016/j.semnephrol.2010.10.004 21266263 PMC3063446

[B33] Mozurkewich E. L. Luke B. Avni M. Wolf F. M. (2000). Working conditions and adverse pregnancy outcome: a meta-analysis. Obstet. Gynecol. 95, 623–635 10.1016/S0029-7844(99)00598-0 10725502

[B34] Nagoshi E. Saini C. Bauer C. Laroche T. Naef F. Schibler U. (2004). Circadian gene expression in individual fibroblasts: cell-autonomous and self-sustained oscillators pass time to daughter cells. Cell 119, 693–705 10.1016/j.cell.2004.11.015 15550250

[B35] Nakahata Y. Kaluzova M. Grimaldi B. Sahar S. Hirayama J. Chen D. (2008). The NAD+-dependent deacetylase SIRT1 modulates CLOCK-mediated chromatin remodeling and circadian control. Cell 134, 329–340 10.1016/j.cell.2008.07.002 18662547 PMC3526943

[B36] Okamura H. Doi M. Yamaguchi Y. Fustin J. M. (2011). Hypertension due to loss of clock: novel insight from the molecular analysis of Cry1/Cry2-deleted mice. Curr. Hypertens. Rep. 13, 103–108 10.1007/s11906-011-0181-3 21286865

[B37] Olofsson P. Poulsen H. (1997). Reversed circadian blood pressure rhythm preserves fetal growth in preeclamptic pregnancy. Eur. J. Obstet. Gynecol. Reprod. Biol. 75, 133–138 10.1016/S0301-2115(97)00099-7 9447364

[B38] Pechere-Bertschi A. Burnier M. (2004). Female sex hormones, salt, and blood pressure regulation. Am. J. Hypertens. 17, 994–1001 10.1016/j.amjhyper.2004.08.009 15485766

[B39] Pennington K. A. Schlitt J. M. Jackson D. L. Schulz L. C. Schust D. J. (2012). Preeclampsia: multiple approaches for a multifactorial disease. Dis. Model Mech. 5, 9–18 10.1242/dmm.008516 22228789 PMC3255538

[B40] Ratajczak C. K. Herzog E. D. Muglia L. J. (2010). Clock gene expression in gravid uterus and extra-embryonic tissues during late gestation in the mouse. Reprod. Fertil. Dev. 22, 743–750 10.1071/RD09243 20450826 PMC3816753

[B41] Redman C. W. Beilin L. J. Bonnar J. (1976). Reversed diurnal blood pressure rhythm in hypertensive pregnancies. Clin. Sci. Mol. Med. Suppl. 3, 687s–689s 1071709 10.1042/cs051687s

[B42] Reischl S. Kramer A. (2011). Kinases and phosphatases in the mammalian circadian clock. FEBS Lett. 585, 1393–1399 10.1016/j.febslet.2011.02.038 21376720

[B43] Reppert S. M. Weaver D. R. (2002). Coordination of circadian timing in mammals. Nature 418, 935–941 10.1038/nature00965 12198538

[B44] Ripperger J. A. Albrecht U. (2012). The circadian clock component PERIOD2: from molecular to cerebral functions. Prog. Brain Res. 199, 233–245 10.1016/B978-0-444-59427-3.00014-9 22877669

[B45] Ripperger J. A. Schibler U. (2006). Rhythmic CLOCK-BMAL1 binding to multiple E-box motifs drives circadian Dbp transcription and chromatin transitions. Nat. Genet. 38, 369–374 10.1038/ng1738 16474407

[B46] Roberge S. Giguere Y. Villa P. Nicolaides K. Vainio M. Forest J. C. (2012a). Early administration of low-dose aspirin for the prevention of severe and mild preeclampsia: a systematic review and meta-analysis. Am. J. Perinatol. 29, 551–556 22495898 10.1055/s-0032-1310527

[B47] Roberge S. Villa P. Nicolaides K. Giguere Y. Vainio M. Bakthi A. (2012b). Early administration of low-dose aspirin for the prevention of preterm and term preeclampsia: a systematic review and meta-analysis. Fetal. Diagn. Ther. 31, 141–146 10.1159/000336662 22441437

[B48] Sattar N. Greer I. A. (2002). Pregnancy complications and maternal cardiovascular risk: opportunities for intervention and screening? BMJ 325, 157–160 10.1136/bmj.325.7356.157 12130616 PMC1123678

[B49] Sawyer M. M. Lipshitz J. Anderson G. D. Dilts P. V. Jr. Halperin L. (1981). Diurnal and short-term variation of blood pressure: comparison of preeclamptic, chronic hypertensive, and normotensive patients. Obstet. Gynecol. 58, 291–296 7266948

[B50] Seligman S. A. (1971). Diurnal blood-pressure variation in pregnancy. J. Obstet. Gynaecol. Br. Commonw. 78, 417–422 10.1111/j.1471-0528.1971.tb01637.x 5558327

[B51] Sibai B. Dekker G. Kupferminc M. (2005). Pre-eclampsia. Lancet 365, 785–799 10.1016/S0140-6736(05)71003-5 15733721

[B52] Sibai B. M. Caritis S. Hauth J. National Institute of Child Health and Human Development Maternal-Fetal Medicine Units Network (2003). What we have learned about preeclampsia. Semin. Perinatol. 27, 239–246 10.1016/S0146-0005(03)00022-3 12889591

[B53] Sibai B. M. Caritis S. N. Thom E. Klebanoff M. McNellis D. Rocco L. (1993). Prevention of preeclampsia with low-dose aspirin in healthy, nulliparous pregnant women. The National Institute of Child Health and Human Development Network of Maternal-Fetal Medicine Units. N. Engl. J. Med. 329, 1213–1218 10.1056/NEJM199310213291701 8413387

[B54] Snoep J. D. Hovens M. M. Pasha S. M. Frolich M. Pijl H. Tamsma J. T. (2009). Time-dependent effects of low-dose aspirin on plasma renin activity, aldosterone, cortisol, and catecholamines. Hypertension 54, 1136–1142 10.1161/HYPERTENSIONAHA.109.134825 19805643

[B55] Stoynev A. G. Penev P. D. Peneva A. V. Cornelissen G. Halberg F. Ikonomov O. C. (1999). Blood pressure and heart rate rhythmicity: differential effects of late pregnancy. Physiol. Behav. 66, 269–275 10.1016/S0031-9384(98)00292-3 10336153

[B56] Straif K. Baan R. Grosse Y. Secretan B. El Ghissassi F. Bouvard V. (2007). Carcinogenicity of shift-work, painting, and fire-fighting. Lancet Oncol. 8, 1065–1066 10.1016/S1470-2045(07)70373-X 19271347

[B57] Tranquilli A. L. Garbati E. Valensise H. Garzetti G. G. Romanini C. (1996). Circadian blood pressure patterns in pregnant women with intrauterine growth retardation. Ann. N. Y. Acad. Sci. 783, 337–339 10.1111/j.1749-6632.1996.tb26738.x 8853663

[B58] Waddell B. J. Wharfe M. D. Crew R. C. Mark P. J. (2012). A rhythmic placenta? Circadian variation, clock genes and placental function. Placenta 33, 533–539 10.1016/j.placenta.2012.03.008 22525887

[B59] Wergeland E. Strand K. (1997). Working conditions and prevalence of pre-eclampsia, Norway 1989. Int. J. Gynaecol. Obstet. 58, 189–196 10.1016/S0020-7292(97)00083-0 9252254

[B60] Wharfe M. D. Mark P. J. Waddell B. J. (2011). Circadian variation in placental and hepatic clock genes in rat pregnancy. Endocrinology 152, 3552–3560 10.1210/en.2011-0081 21771885

